# Characterization of Cell Death Induced by Imine Analogs of Trans-Resveratrol: Induction of Mitochondrial Dysfunction and Overproduction of Reactive Oxygen Species Leading to, or Not, Apoptosis without the Increase in the S-Phase of the Cell Cycle

**DOI:** 10.3390/molecules28073178

**Published:** 2023-04-03

**Authors:** Mohamed Ksila, Imen Ghzaiel, Vivien Pires, Taoufik Ghrairi, Olfa Masmoudi-Kouki, Norbert Latruffe, Dominique Vervandier-Fasseur, Anne Vejux, Gérard Lizard

**Affiliations:** 1 Team ‘Biochemistry of the Peroxisome, Inflammation and Lipid Metabolism’ EA7270/Inserm, University of Bourgogne, 21000 Dijon, France; mohamedksila44@gmail.com (M.K.); imenghzaiel93@gmail.com (I.G.); vivien.pires@u-bourgogne.fr (V.P.); norbert.latruffe@u-bourgogne.fr (N.L.); anne.vejux@u-bourgogne.fr (A.V.); 2 Laboratory of Neurophysiology, Cellular Physiopathology and Valorisation of Biomolecules, (LR18ES03), Department of Biology, Faculty of Sciences, University Tunis El Manar, Tunis 2092, Tunisia; taoufik.ghrairi@fst.utm.tn (T.G.); olfa.masmoudi@fst.utm.tn (O.M.-K.); 3 Team OCS, Institute of Molecular Chemistry (ICMUB UMR CNRS 6302), University of Bourgogne, 21000 Dijon, France; dominique.vervandier-fasseur@u-bourgogne.fr

**Keywords:** apoptosis, aza-stilbenes, cell death, cell cycle, flow cytometry, imine analogs of *trans*-resveratrol, mitochondria, oxidative stress, *trans*-resveratrol

## Abstract

*Trans*-resveratrol (RSV) is a non-flavonoid polyphenol (stilbene) with numerous biological activities, such as anti-tumor activities. However, RSV is rapidly metabolized, which limits its therapeutic use. The availability of RSV analogues with similar activities for use in vivo is therefore a major challenge. For this purpose, several isomeric analogues of RSV, aza-stilbenes (AZA-ST 1a–g), were synthesized, and their toxicities were characterized and compared to those of RSV on murine N2a neuronal cells using especially flow cytometric methods. All AZA-ST 1a–g have an inhibitory concentration 50 (IC50) between 11.3 and 25 µM when determined by the crystal violet assay, while that of RSV is 14.5 µM. This led to the characterization of AZA-ST 1a–g—induced cell death, compared to RSV, using three concentrations encompassing the IC50s (6.25, 12.5 and 25 µM). For AZA-ST 1a–g and RSV, an increase in plasma membrane permeability to propidium iodide was observed, and the proportion of cells with depolarized mitochondria measured with DiOC_6_(3) was increased. An overproduction of reactive oxygen species (ROS) was also observed on whole cells and at the mitochondrial level using dihydroethidium and MitoSox Red, respectively. However, only RSV induced a mode of cell death by apoptosis associated with a marked increase in the proportion of cells with condensed and/or fragmented nuclei (12.5 µM: 22 ± 9%; 25 µM: 80 ± 10%) identified after staining with Hoechst 33342 and which are characteristic of apoptotic cells. With AZA-ST, a slight but significant increase in the percentage of apoptotic cells was only detected with AZA-ST 1b (25 µM: 17 ± 1%) and AZA-ST 1d (25 µM: 26 ± 4%). Furthermore, only RSV induced significant cell cycle modifications associated with an increase in the percentage of cells in the S phase. Thus, AZA-ST 1a–g—induced cell death is characterized by an alteration of the plasma membrane, an induction of mitochondrial depolarization (loss of ΔΨm), and an overproduction of ROS, which may or may not result in a weak induction of apoptosis without modification of the distribution of the cells in the different phases of the cell cycle.

## 1. Introduction

**Figure 1:**
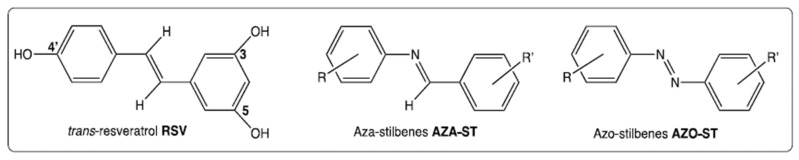
Structure of *trans*-resveratrol (RSV), aza-stilbenes (AZA-ST), and azo-stilbenes (AZO-ST) [15].

Polyphenols are molecules present in many plants and are major components of the Mediterranean diet, known for its health benefits [1,2]. These molecules are subdivided into two groups: flavonoids and non-flavonoids [3]. Among the latter, *trans*-resveratrol (RSV), which belongs to the stilbene subclass, is one of the most studied polyphenols because of its numerous biological activities, including antioxidant [4], anti-inflammatory [5], anti-tumor [6], and anti-ageing activities [7]. RSV has also shown neurotrophic activities in the context of Parkinson’s disease [8] and cytoprotective activities against oxidized cholesterol derivatives (oxysterols, especially 7-ketocholesterol, mainly formed by cholesterol autoxidation [9]), often found in increased amounts in patients with age-related diseases (cardiovascular diseases, Alzheimer’s disease, and age-related macular degeneration) [10]. However, one of the major problems observed with RSV is its rapid catabolism by the intestinal microbiota and at the blood and tissue levels, leading to the formation of inactive metabolites that limit its therapeutic use [11]. If increasing the bioavailability of RSV by using galenic solutions (micro- and nano-encapsulation, functionalized nanoparticles) fails, it is also possible to synthesize analogues of RSV, which requires ensuring that the latter retain biological activities close to those of RSV or even are more effective. Currently, several synthetic derivatives of RSV have been developed that retain the original stilbene structure and preserve its water-soluble character. Moreover, the C=C bond may be replaced with an isosteric fragment as an aromatic heterocycle [12] or with a C=N bond or an N=N bond to provide aza-stilbenes (AZA-ST) [13] and azo-stilbenes [14], respectively [15] (Figure 1).

**Figure 2:**
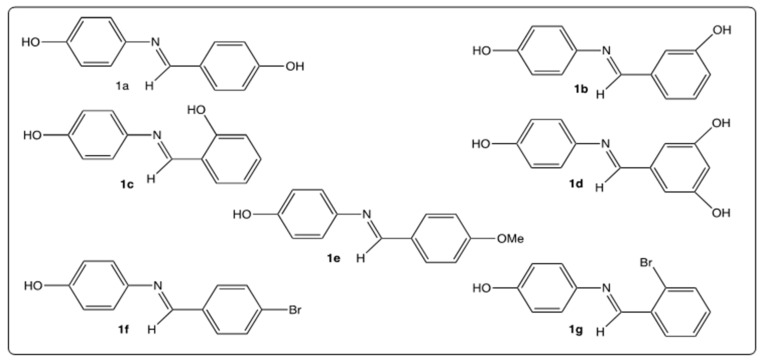
Structures of AZA-ST 1a–g [15].

We previously reported the synthesis of a series of seven imine analogs of RSV (AZA-ST 1a–g) (Figure 2) by an easy and low-cost green chemistry procedure [15]. In this study, the different AZA-STs were chemically characterized [15]. We showed that all AZA-ST 1a–g synthesized could be distinguished from RSV based on their cytotoxic and antioxidant characteristics. In this previous study, the cytotoxicity was evaluated with the sulforhodamine 101 (SR101), the fluorescein diacetate (FDA), and the DiOC_6_(3) assays, but the type of cell death was not characterized. However, the antioxidant activity of AZA-ST 1a–g was quantified with the KRL kit (Radicaux Libres) assay, the DPPH (2,2′-diphenyl-1-picrylhydrazyl radical) assay, the FRAP (Ferric Reducing Antioxidant Power) assay, and the PAOT (Pouvoir AntiOxidant Total) score [15]. Based on their cytotoxic and antioxidant characteristics, the AZA-ST 1a–g was distinguishable from the RSV. Interestingly, the presence of one or more hydroxyl groups and their position on the aromatic cycle provide more or less strong antioxidant activities to AZA-STs compared to RSV [16]. At the moment, aza- and azo-stilbenes have been reported to oppose the aggregation of β-amyloids considered neurotoxic factors in Alzheimer’s disease [17], and to inhibit cholinesterase [18]. Inhibition of growth through autophagy has been shown by aza- and azo-stibenes against MDA-MB-231 and T47D breast cancer cell lines [19].

The aim of the present study was to (i) compare the toxicity of AZA-ST 1a–g to RSV on murine N2a neuronal cells and (ii) characterize the mode of cell death induced by AZA-ST 1a–g and RSV on N2a cells. The inhibitory concentrations (IC50) were determined with the crystal violet assay [20]. The cell death was characterized by different criteria evaluated by flow cytometry: plasma membrane permeability evaluated with propidium iodide (PI), transmembrane mitochondrial potential (ΔΨm) measured with DiOC_6_(3), ROS overproduction measured on whole cells after staining with dihydroethidium (DHE) and at the mitochondrial level with MitoSox Red [21], and distribution of the cells in the different phases of the cell cycle [22]. In addition, nuclear morphology was determined after nuclear staining with Hoechst 33442, which allows to distinguish between viable, apoptotic, and necrotic/oncotic cells [23]. The different parameters studied permit to distinguish AZA-ST 1a–g—induced cell death from RSV-induced cell death.

## 2. Results

### 2.1. Cell Growth Evaluation by Crystal Violet Assay and Cell Viability Evaluation by Propidium Iodide Staining

**Figure 3:**
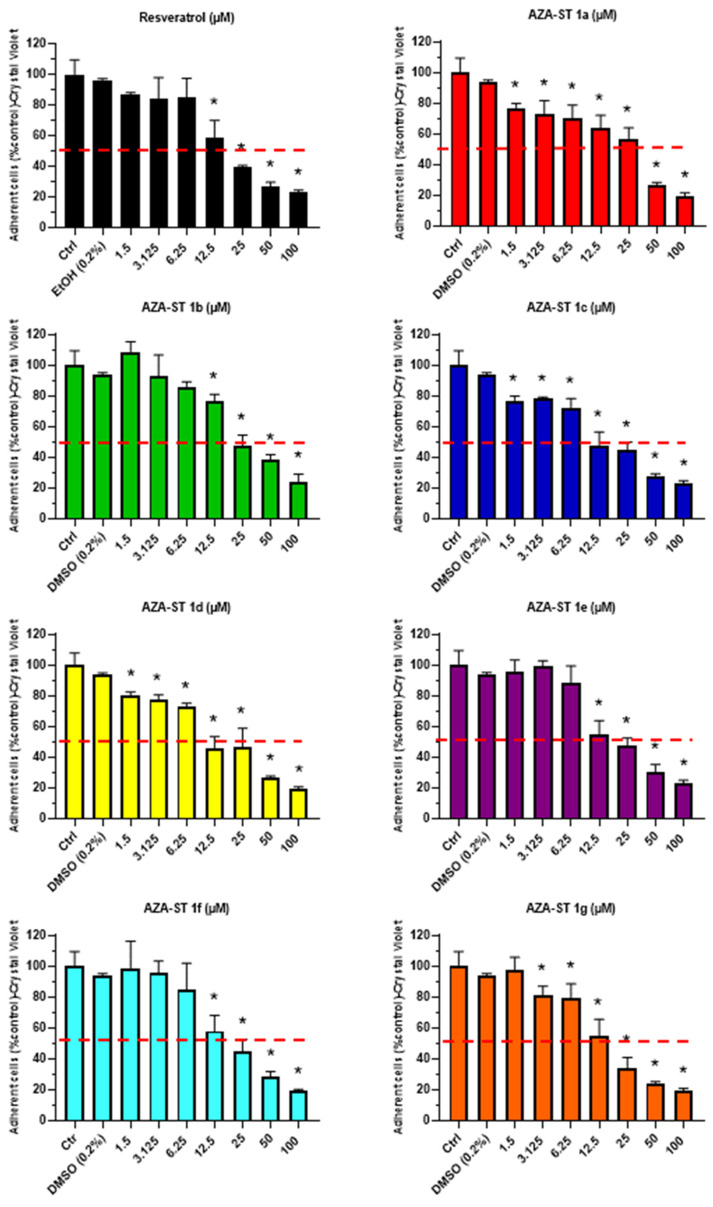
Crystal violet assay evaluation of the effects of AZA-ST 1a–g and resveratrol on cell growth. N2a cells were incubated for 24 h with or without AZA-ST 1a–g or resveratrol (*trans*-resveratrol) in a range of concentrations from 1.5 to 100 μM. The dotted red line makes it possible to evaluate the value of the concentration (or the range of concentrations) reducing cell growth by 50% (IC50); IC50 values were calculated with GraphPad. Vehicle of AZA-ST: DMSO (0.2%) [15]. The data are the mean ± standard deviation (SD) of three independent experiments performed in triplicate. The comparison of the data was performed with an ANOVA test followed by a Bonferroni test. (*): *p* ≤ 0.05 was considered statistically significant.

**Figure 4:**
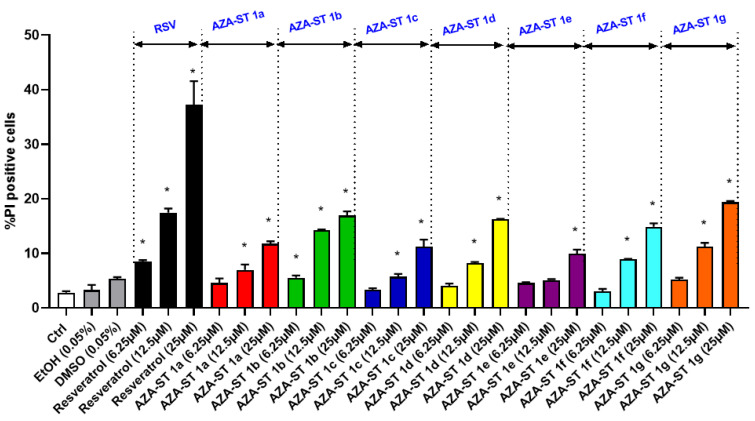
Flow cytometric evaluation of the effects of AZA-ST 1a–g and resveratrol on plasma membrane damages and cell viability by staining with propidium iodide (PI). N2a cells were incubated for 24 h with or without AZA-ST 1a–g or resveratrol (*trans*-resveratrol) at concentrations in the range of the IC50s (6.25, 12.5 and 25 µM). The data are the mean ± standard deviation (SD) of three independent experiments performed in triplicate. The comparison of the data was performed with an ANOVA test followed by a Bonferroni test. (*): *p* ≤ 0.05 was considered statistically significant. Vehicles (Ethanol (EtOH) for RSV; DMSO for AZA-ST) [15]: EtOH (0.05%) and DMSO (0.05%); the highest concentrations of vehicles were only analyzed.

The impact of AZA-ST 1a–g and *trans*-resveratrol (RSV) on cell growth was evaluated at the different concentrations (i.e., 1.5, 3.125, 6.25, 12.5, 25, 50, and 100 µM) using the crystal violet assay, which permits the quantification of adherent cells. The calculated IC50 values obtained with the crystal violet assay were in the same range of order and were the following: AZA-ST 1c: 11.30 ± 0.57; AZA-ST 1d: 11.48 ± 0.57; AZA-ST 1g: 11.75 ± 0.58; AZA-ST 1e: 12.43 ± 0.62; AZA-ST 1f: 13.95 ± 0.69; RSV: 14.95 ± 0.74; AZA-ST 1b: 19.49 ± 0.97; AZA-ST 1a: 25.00 ± 1.25 (Figure 3). This inhibition of cell growth, at concentrations inclosing the IC50s (6.25, 12.5 and 25 µM), is associated with an increase in permeability to propidium iodide (PI), which is considered a criteria for cell death [24], and which also indicates that AZA-ST 1a–g—and RSV-induced cell death is associated with an alteration of the plasma membrane [23]. Thus, a more or less pronounced concentration-dependent increase in PI-positive cells (cells with damaged plasma membranes considered to be dead cells) was observed both with AZA-ST 1a–g and RSV (Figure 4).

### 2.2. Measurement of Transmembrane Mitochondrial Potential by Staining with DiOC_6_(3)

**Figure 5:**
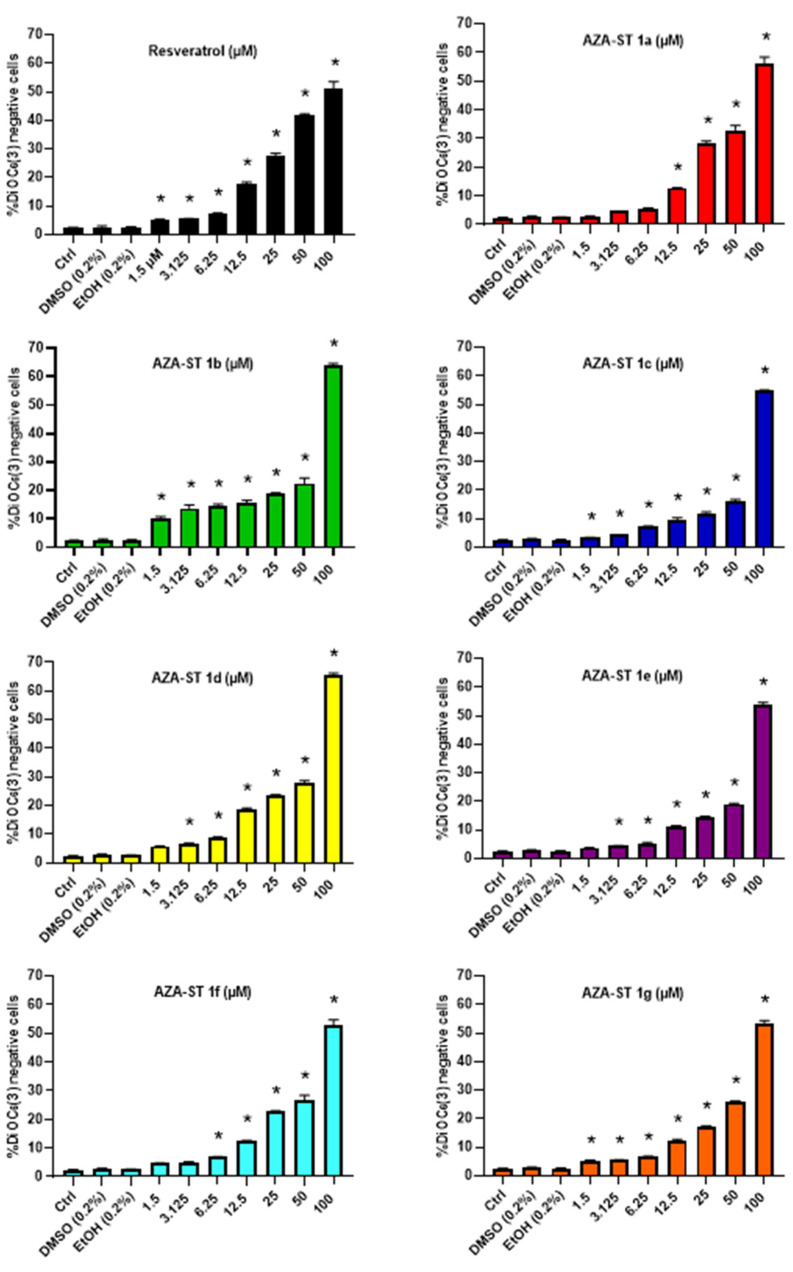
Flow cytometric evaluation of the effects of AZA-ST 1a–g and resveratrol on transmembrane mitochondrial potential (ΔΨm) by staining with DiOC_6_(3). N2a cells were incubated for 24 h with or without AZA-ST 1a–g or resveratrol (RSV; *trans*-resveratrol) at concentrations inclosing the IC50s (6.25, 12.5 and 25 µM). Vehicles (Ethanol (EtOH) for RSV; DMSO for AZA-ST) [15]: EtOH (0.2%) and DMSO (0.2%); the highest concentrations of vehicles were only analyzed. The data are the mean ± standard deviation (SD) of three independent experiments performed in triplicate. The comparison of the data was performed with an ANOVA test followed by a Bonferroni test. (*): *p* ≤ 0.05 was considered statistically significant.

As it is well established that RSV-induced cell death is associated with a loss of transmembrane mitochondrial potential (ΔΨm) on different cell types, the ability of AZA-ST 1a–g and resveratrol (RSV) to trigger a loss of ΔΨm on N2a cells was measured by flow cytometry after staining with DiOC_6_(3). In these conditions, at concentrations inclosing the IC50s (6.25, 12.5 and 25 µM), a more or less pronounced concentration-dependent increase of the percentage of cells with depolarized mitochondria (DiOC_6_(3) negative cells) was observed both with AZA-ST 1a–g and RSV (Figure 5).

### 2.3. Measuring Reactive Oxygen Species Production on Whole Cells at the Mitochondrial Level with Dihydroethidium and MitoSox Red

**Figure 6:**
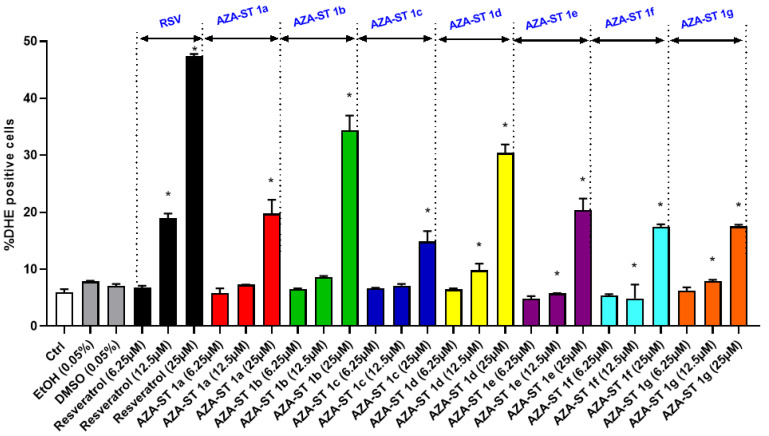
Flow cytometric evaluation of the effects of AZA-ST 1a–g and resveratrol on ROS overproduction in whole cells by staining with dihydroethidium (DHE). N2a cells were incubated for 24 h with or without AZA-ST 1a–g or resveratrol (RSV; *trans*-resveratrol) at concentrations inclosing the IC50s (6.25, 12.5 and 25 µM). Vehicles (Ethanol (EtOH) for RSV; DMSO for AZA-ST) [15]: Ethanol (EtOH; 0.05%) and DMSO (0.05%); the highest concentrations of vehicles were only analyzed. The data are the mean ± standard deviation (SD) of three independent experiments performed in triplicate. The comparison of the data was performed with an ANOVA test followed by a Bonferroni test. (*): *p* ≤ 0.05 was considered statistically significant.

It is well known that oxidative stress can be associated with cell death and can contribute to cell damage induction in some molecules (lipids, proteins, and DNA) [25]. Therefore, the ability of AZA-ST 1a–g and RSV to trigger ROS overproduction on whole cells at the mitochondrial level was evaluated on N2a cells. At three concentrations in between the IC50s (6.25, 12.5 and 25 µM), an overproduction of ROS was observed on whole cells with AZA-ST 1a–g and RSV; the highest percentages of cells affected by ROS overproduction, which were identified by staining with dihydroethidium (DHE positive cells), were observed under treatment with RSV and were in the following order at 25 µM: RSV > AZA-ST 1b > AZA-ST 1d > AZA-ST 1a–e > AZA-ST 1f–g > AZA-ST 1c (Figure 6).

**Figure 7:**
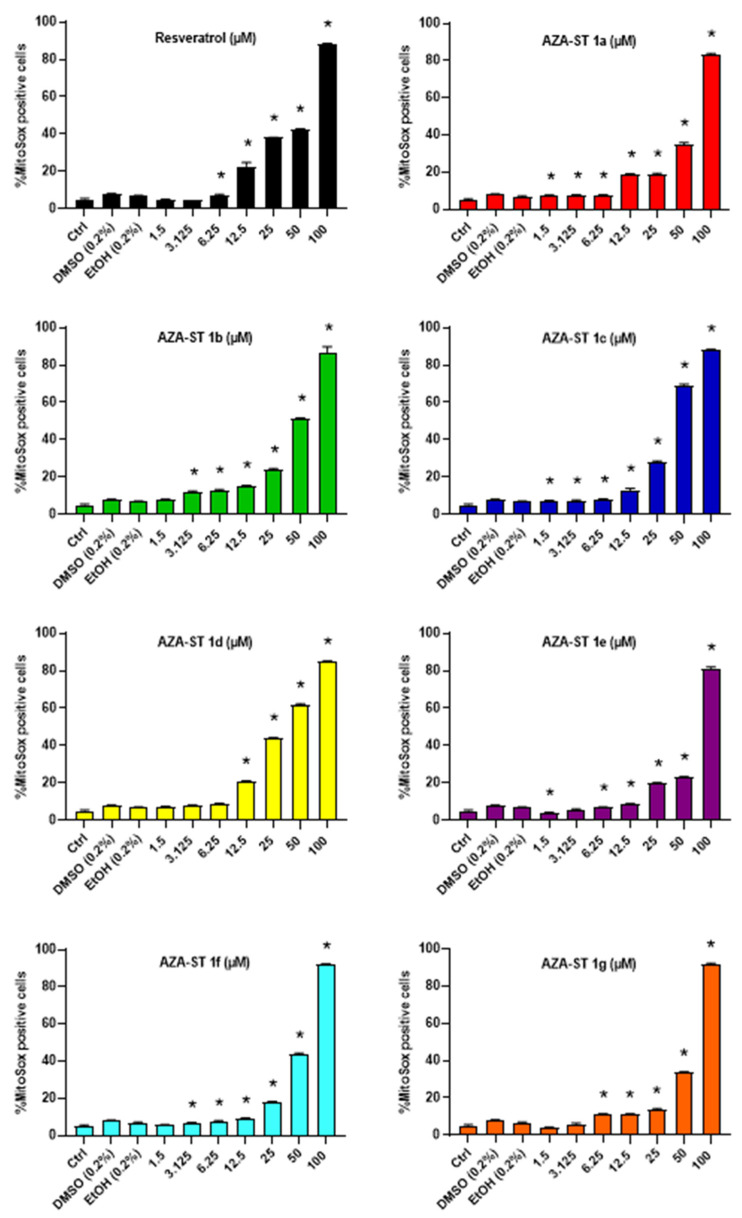
Flow cytometric evaluation of the effects of AZA-ST 1a–g and resveratrol on ROS overproduction at the mitochondrial level by staining with MitoSox Red. N2a cells were incubated for 24 h with or without AZA-ST 1a–g or resveratrol (RSV; *trans*-resveratrol) at concentrations from 1.5 to 100 µM. Vehicles (Ethanol (EtOH) for RSV; DMSO for AZA-ST) [15]: Ethanol (EtOH; 0.2%) and DMSO (0.2%); the highest concentrations of vehicles were the only ones analyzed. The data are the mean ± standard deviation (SD) of three independent experiments performed in triplicate. The comparison of the data was performed with an ANOVA test followed by a Bonferroni test. (*): *p* ≤ 0.05 was considered statistically significant.

In addition, ROS overproduction was also measured at the mitochondrial level after staining with MitoSox Red in a range of concentrations from 1.5 to 100 µM. A more or less pronounced concentration-dependent increase in the percentage of cells with an overproduction of ROS occurring at the mitochondrial level (MitoSox positive cells) was observed both with AZA-ST 1a–g and RSV (Figure 7).

### 2.4. Microscopical Characterization of Cell Death after Nuclei Staining with Hoechst 33342

**Figure 8:**
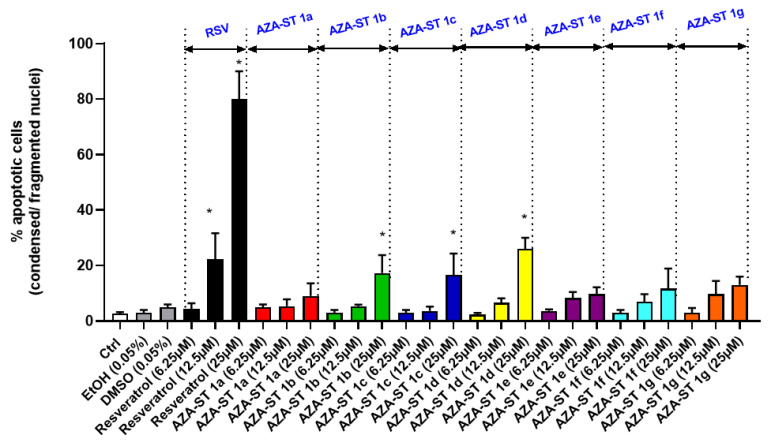
Quantification of apoptotic cells by fluorescence microscopy after nuclei staining with Hoechst 33342: effects of AZA-ST 1a–g and resveratrol on apoptosis induction. N2a cells were incubated for 24 h with or without AZA-ST 1a–g or resveratrol (RSV; *trans*-resveratrol) at concentrations inclosing the IC50s (6.25, 12.5 and 25 µM). Vehicles (Ethanol (EtOH) for RSV; DMSO for AZA-ST) [4]: Ethanol (EtOH; 0.05%) and DMSO (0.05%); the highest concentrations of vehicles were only analyzed. The data are the mean ± standard deviation (SD) of three independent experiments performed in triplicate. The comparison of the data was performed with an ANOVA test followed by a Bonferroni test. (*): *p* ≤ 0.05 was considered statistically significant.

RSV is known to induce apoptosis in different cell types. This polyphenol can also activate alternative pathways leading to cell death, such as those leading to autophagy, senescence, or mitotic catastrophe [26]. It was therefore important to determine whether AZA-ST 1a–g induces or not the same type of cell death as RSV on N2a cells. Cell death was morphologically characterized by fluorescence microscopy after nuclei were stained with Hoechst 33342 [23]. Indeed, this microscopical method is suitable to easily identify apoptotic cells, which are characterized by condensed and/or fragmented nuclei [23,27]. In these conditions, at the three concentrations corresponding to the IC50s (6.25, 12.5 and 25 µM), only RSV induced a marked mode of cell death by apoptosis associated with a strong increase in the proportion of cells with condensed and/or fragmented nuclei (12.5 µM: 22 ± 9%; 25 µM: 80 ± 10%) (Figure 8). With AZA-ST, a slight but significant increase in the percentage of apoptotic cells was only detected with AZA-ST 1b (25 µM: 17 ± 1%) and AZA-ST 1d (25 µM: 26 ± 4%) (Figure 8). Slight and not significant increases in apoptotic cells were observed with AZA-ST 1a, 1c, 1e, 1f, and 1g (Figure 8).

### 2.5. Flow Cytometric Analysis of the Impact of Aza-Stilbenes and Resveratrol on the Repartition of Cells in Different Phases of the Cell Cycle

**Figure 9:**
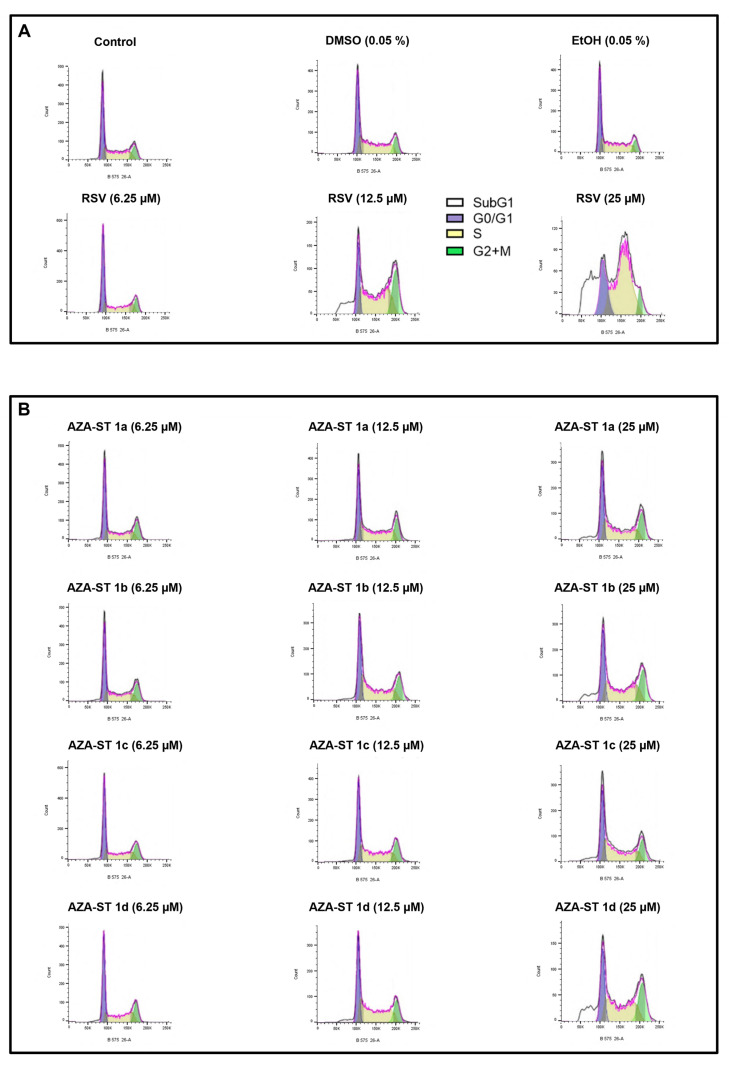

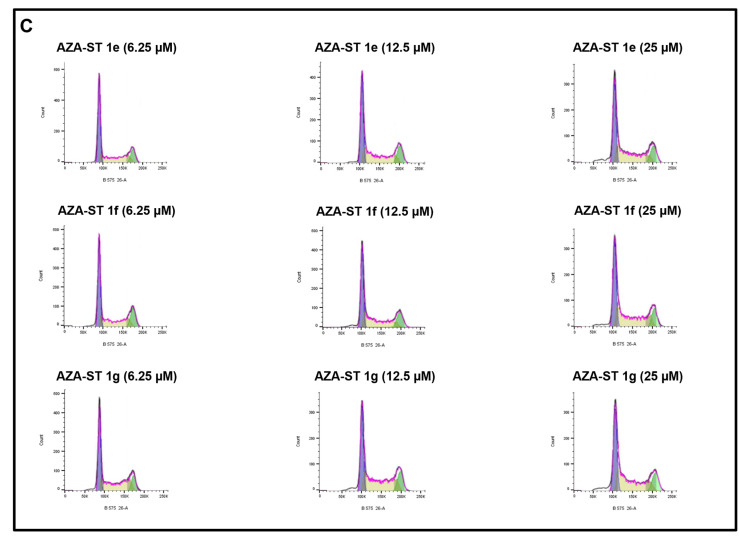
Flow cytometric analysis of the distribution of the cells in the different phases of the cell cycle: effects of AZA-ST 1a–g and resveratrol. N2a cells were incubated for 24 h with or without AZA-ST 1a–g or resveratrol (RSV; *trans*-resveratrol) at concentrations inclosing the IC50s (6.25, 12.5 and 25 µM). (**A**): Control, vehicles (DMSO and EtOH); (**B**): AZA-ST 1a–d; (**C**): AZA-ST-1e–g. Vehicles (Ethanol (EtOH) for RSV; DMSO for AZA-ST) [15]: Ethanol (EtOH; 0.05%) and DMSO (0.05%); only the highest concentrations of vehicles were analyzed. White: SubG1; purple: G0/G1 phase; yellow: S phase; green: (G2 + M) phase.

**Figure 10:**
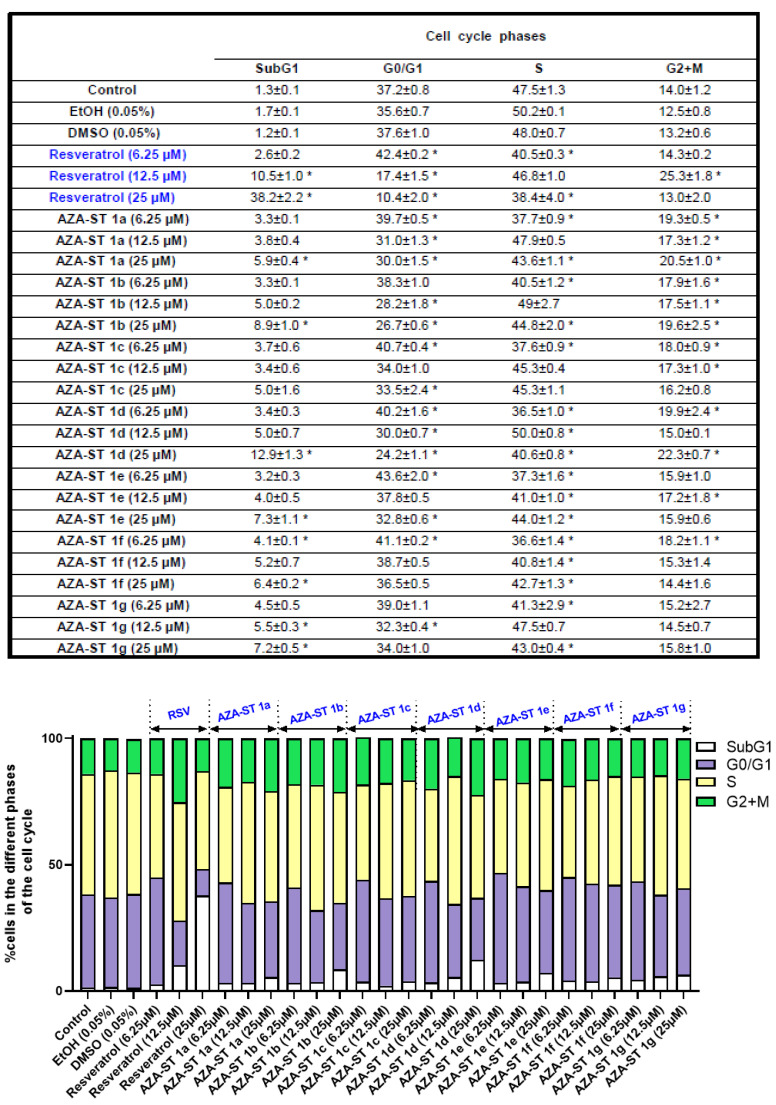
Quantification of the cells in SubG1 and in the different phases (G0/G1, S and (G2 + M)) of the cell cycle. N2a cells were incubated for 24 h with or without AZA-ST 1a–g or resveratrol (RSV; *trans*-resveratrol) at concentrations inclosing the IC50s (6.25, 12.5 and 25 µM). Vehicles (Ethanol (EtOH) for RSV; DMSO for AZA-ST) [15]: Ethanol (EtOH; 0.05%) and DMSO (0.05%); only the highest concentrations of vehicles were analyzed. White: SubG1; purple: G0/G1 phase; yellow: S phase; green: (G2 + M) phase. The data are the mean ± standard deviation (SD) of three independent experiments performed in triplicate. The comparison of the data was performed with an ANOVA test followed by a Bonferroni test. (*): *p* ≤ 0.05 was considered statistically significant.

Resveratrol has been described to induce a mode of cell death by apoptosis associated with an increase in the percentage of cells in the S phase of the cell cycle [28,29]. In addition, it has been well established that apoptosis can, in some cases, be associated with a population of cells in SubG1, resulting from inter-nucleosomal DNA fragmentation, when cell cycle analysis is performed by flow cytometry after PI staining [30,31]. The analysis of the cell cycle by flow cytometry and PI staining, which was realized on AZA-ST 1a–g and RSV-treated cells, therefore had two objectives: to define the percentage of cells in the different phases of the cell cycle and to demonstrate the presence or absence of a Sub-G1 peak. Cell cycle analysis was carried out at the IC50 concentrations of 6.25, 12.5, and 25µM (Figure 9 and Figure 10). 

In agreement with previous studies, important modifications of the distribution of the cells in the different phases of the cell cycle were observed with RSV: a strong increase of cells in the S-phase was shown, as well as a SubG1 peak, which is an apoptotic criterion.

Under treatment with AZA-ST 1a–g, the most important modifications were observed with AZA-ST 1b and 1d. A SubG1 peak, which supports the induction of apoptosis in agreement with the presence of condensed and/or fragmented nuclei, was observed (Figure 9 and Figure 10). With the other aza-stilbenes, no modification or slight modification of the distribution of the cells in the different phases of the cell cycle was observed (Figure 9 and Figure 10).

## 3. Discussion

*Trans*-resveratrol (RSV) is a polyphenol with multiple biological activities that can vary, particularly in vitro, depending on the concentrations used. At low concentrations, not exceeding 20 µM, RSV has antioxidant activities, which can involve nuclear factor erythroid 2-related factor 2 (Nrf2) signaling through blockage of Kelch-like ECH Associated Protein 1 (Keap1) [32] and anti-inflammatory properties [33]. Regarding antioxidant activities, RSV, like all polyphenols, has phenolic hydroxyl groups, Ar (aromatic)—OH, which can supply H to L-OO• peroxyl radicals and thus neutralize them in the form of L-OOH hydroxides [34]. RSV is also a potent senolytic compound that prevents the senescence involved in aging [35]. RSV also has neurotrophic activities (antioxidant and differentiating activities) on hydrogen peroxide (H_2_O_2_)—treated N2a cells [8,36]. On N2a cells, RSV also prevents cytotoxicity induced by 7KC and 7β-OHC, two oxysterols whose concentrations are strongly increased in several age-related diseases: cardiovascular diseases, neurodegenerative diseases, age-related macular degeneration (AMD), osteoporosis, and sarcopenia [25,37]. At high concentrations, from 50 to 100 µM, RSV has potent anti-tumor activities by inducing death by apoptosis [26]. In addition, in vivo, RSV increases lifespan in *Caenorhabditis elegans, Drosophila melanogaster*, and mice by stimulating the AMPK pathway and activating sirtuins [38,39,40,41]. One of the first studies of the effect of RSV on the lifespan was realized on a small, short-lived fish, the *Nothobranchius furzei* [42]. However, one of the main weaknesses of RSV is its rapid catabolism, both at the plasma level and by various bacteria at the intestinal level [11].

The synthesis of structural analogues is one of the possible strategies for the preservation of important RSV health properties and to oppose its catabolism. This led us to the synthesis of aza-stilbenes [15]. These molecules have shown cytotoxic activities on N2a cells in vitro and high anti-free radical properties when measured by various biochemical tests (DPPH, FRAP, and PAOT) and with the KRL assay performed in the presence of AAPH on red blood cells [15]. It is indeed possible that these compounds could be good candidates for anti-tumor activities in vivo and/or to limit the occurrence of age-related diseases frequently associated with a rupture of RedOx homeostasis leading to ROS overproduction, lipid peroxidation, protein carbonylation, DNA damage, and subsequently numerous cell damages. However, before realizing in vivo studies with AZA-STs, it is important to know more about their in vitro biological activities compared with those of RSV. Thus, in the present study performed on murine neuronal N2a cells, the impact of AZA-STs and RSV on ROS overproduction was studied, and the type of cell death induced by these molecules was also characterized. The synthesised AZA-STs, like RSV, induce cell death with IC50s in the range of 10 µM, except for ASA-ST 1a, whose IC50 was 25 µM. Moreover, all the AZA-STs studied induce a mode of cell death associated with oxidative stress. However, unlike RSV, which is a potent inducer of apoptosis, the AZA-STs are either weak inducers of apoptosis, such as AZA-ST 1b and 1d, or do not induce this type of cell death. Furthermore, while RSV induces apoptosis associated with an increase in the percentage of cells in the S phase of the cell cycle, no significant change in the distribution of the cells in the different phases of the cycle was observed with all AZA-STs studied.

Thus, with the crystal violet assay, which measures the amount of adherent cells after nuclei staining, the toxicity of the different synthesised AZA-STs was in the range of that of RSV (10 µM), with the exception of AZA-ST 1a, whose IC50 was 25 µM. Differences in IC50 between RSV and AZA-ST (6.25 to 50 µM) were also observed when the cytotoxic effects were measured either by the FDA assay, which evaluates esterase activity, or by the SR101 assay, which allows determining the amount of adherent cells while taking into account the total amount of protein [20,43]. Although these different tests (the crystal violet, FDA, and SR101 assays) measure different parameters, the IC50 values found are of the same order of magnitude. Noteworthy, like RSV, the toxicity associated with AZA-ST is accompanied by a decrease in adherent cells, which can be attributed to a decrease in cell multiplication but also to a loss of cell adhesion also observed with quercetin and apigenin [8]. This toxicity is also accompanied by a decrease in esterase activity, supporting the alteration of the plasma membrane. These different effects, which are well documented for RSV, are therefore similar with AZA-STs.

Furthermore, the toxicity associated with AZA-STs and RSV leads to an increase in the permeability of cells to propidium iodide (PI), which is not only a criterion of cell death but also reflects a strong alteration of the plasma membrane [23,24]. These data agree with those obtained with the FDA test. The causes of this alteration of the plasma membrane could be due to oxidative stress, as the latter is known to alter the lipid components of the plasma membrane. This hypothesis agrees with the results obtained. Indeed, AZA-STs are all powerful inducers of oxidative stress, like RSV. In N2a cells, these molecules increase ROS overproduction on whole cells when measured by flow cytometry using dihydroethidium (DHE) staining, but also at the mitochondrial level when measured by flow cytometry after staining with MitoSox Red. The activation of oxidative stress could therefore be a key event leading to cell death under treatment with AZA-STs and RSV. At the mitochondrial level, a drop in transmembrane potential (ΔΨm) is also observed with the fluorochrome DiOC_6_(3), which is widely used in cell death studies due to its physicochemical properties (cationic and lipophilic dye that mainly accumulates in the mitochondrial membrane proportionally to the ΔΨm value) that make it a reliable tool for measuring transmembrane mitochondrial potential. In the cascade of events leading to cell death under treatment with AZA-STs, it will be further important to specify the chronology between oxidative stress and mitochondrial dysfunctions. Nevertheless, whatever the type of cell death, it is well established that the drop in ΔΨm represents a decisive and irreversible event in cell death induction [44].

In the case of RSV, this fall in ΔΨm is known to activate apoptosis. This is confirmed in the present study performed on N2a cells. In contrast, in the same cells treated with AZA-STs, a non-apoptotic mode of death was induced. With AZA-ST 1a, 1c, 1e, 1f, and 1g, a non-apoptotic cell death was observed. Indeed, compared to untreated cells and vehicle control cells, no increase in cells with condensed and/or fragmented nuclei (typical of apoptotic cells) was observed, and no SubG1 peak, considered an apoptotic criterion, was found. It is known that the SubG1 peak contains morphologically apoptotic cells with inter-nucleosomal fragmented DNA [30,45]. However, with AZA-ST 1b and 1d, a slight but significant increase in apoptotic cells was observed compared to untreated cells (the control) and vehicle-treated cells. These data support a mixed type of death with AZA-ST 1b and 1d: indeed, morphologically apoptotic cells represent only one third of dead cells measured by PI staining, which allows to determine the total number of dead cells. Interestingly, under treatment with AZA-ST 1b and 1d, as well as in the presence of RSV, which are pro-apoptotic molecules, apoptosis was simultaneously characterized by the presence of cells with condensed and/or fragmented nuclei and by a SubG1 peak revealed by flow cytometry. The simultaneous presence of these two apoptotic criteria is not systematic with pro-apoptotic drugs [31]. However, only RSV-induced cell death was associated with an increase in the percentage of cells in the S phase of the cell cycle. Whatever the AZA-ST considered, no modification of the distribution of the cells in the different phases of the cell cycle was observed. With AZA-STs, at the opposite of RSV, an effect on cyclin-dependent kinases (CDKs) [46] or cyclin-dependent kinase inhibitors (CDKIs) [47] involved in the regulation of cell cycle is therefore unlikely.

## 4. Materials and Methods

### 4.1. Chemistry

Aza-stilbenes were synthesized by a one-step condensation reaction between aromatic aldehydes and primary aromatics, as we previously described [15]. For the chemical characterization of all imine analogs synthesized (AZA-ST 1a–g), we have carried out 1H, COSY, and NOESY NMR experiments of all compounds to assign chemical shifts of aromatic protons as well as 13C, jmod, HSQC, and HMBC NMR experiments to highlight coupling between the 1H and 13C nuclei [15]. The 1H (500 MHz), 13C (126 MHz) NMR, and HMBC-NMR spectra of the seven aza-stilbenes synthesized and of *trans*-resveratrol have been realized [15].

### 4.2. Cell Culture and Treatments

The mouse neuro-2a (N2a) neuroblastoma cell line (Ref: CCL-131, American Type Culture Collection (ATCC), Manassas, VA, USA) was cultured as previously described [15]. The cells were cultured in Dulbecco’s modified Eagle medium (DMEM, Lonza, Amboise, France), which contains 10% (*v/v*) of heat-inactivated fetal bovine serum (FBS) (Pan Biotech, Aidenbach, Germany) and 1% (*v/v*) of penicillin (100 U/mL)/streptomycin (100 mg/mL) (Pan Biotech). The cells were incubated at 37 °C in a humidified atmosphere (5% CO_2_, 95% air) and passaged twice a week. The cells were seeded at 60,000 cells per well containing 1 mL of DMEM supplemented with 10% (*v/v*) heat-inactivated FBS and 1% antibiotics (penicillin, streptomycin) in 24-well plates (FALCON, Becton Dickinson, Franklin Lakes, NJ, USA). The stock solutions of resveratrol (RSV; *trans*-resveratrol) and of imine analogs (aza-stilbenes (AZA-ST) 1a–g) were prepared as follows: *trans*-resveratrol (RSV) (reference of the product: 501-36-0; purity 99%; Sigma-Aldrich, St. Quentin-Fallavier, France) was prepared at 50 mM in absolute ethanol (EtOH; 98%; Carlo Erba Reagents, Val de Reuil, France); AZA-ST 1a–g was prepared at 50 mM in dimethyl sulfoxide (DMSO; Sigma-Aldrich, St Quentin-Fallavier, France). In order to evaluate the effects of AZA-ST 1a–g on N2a cells compared to RSV, the growth medium was removed after 24 h of culture, and the N2a cells were incubated either with resveratrol or AZA-ST 1a–g at various concentrations ranging from 1.5 to 100 µM for 48 h. The highest concentration of 100 µM is obtained by diluting 16 µL of stock solution (RSV or AZA-ST; stock solution at 50 mM) in 8 mL of culture medium. Concentrations of 100 to 1.5 µM are obtained by successive dilution in a two-by-two cascade in culture medium. The effects of vehicles (DMSO, ethanol (ETOH)) were evaluated at their highest concentration (0.2% *v/v* when the highest concentrations of RSV and AZA-ST used were 100 µM; 0.05% *v/v* when the highest concentrations of RSV and AZA-ST used were 25 µM). RSV was prepared at 50 mM in absolute ethanol (EtOH 98%). Therefore, when RSV is used at 25 µM, the final concentration of EtOH is around 0.05% (0.049%); when RSV is used at 100 µM, the final concentration of EtOH is around 0.2% (0.196%). AZA-ST were prepared at 50 mM in DMSO (pure, 100%). Therefore, when AZA-ST are used at 25 µM, the final concentration of DMSO is 0.05%; when AZA-ST are used at 100 µM, the final concentration of DMSO is 0.2%.

### 4.3. Staining with Crystal Violet

The quantification of adherent cells was estimated by staining with crystal violet [20]. Cells were seeded in triplicates in 6-well plates and cultured without or with imine analogs of *trans*-resveratrol (aza-stilbenes) or with *trans*-resveratrol (RSV: 1.5, 3.125, 6.25, 12.5, 25, 50, and 100 µM) for 48 h. At the end of treatment, cells were washed with PBS, stained with crystal violet for 5 min (Sigma-Aldrich), and then rinsed with water. Absorbance was read at 570 nm after extraction of the dye with 0.1 mol/L sodium citrate in 50% ethanol.

### 4.4. Evaluation of Cell Morphology by Phase Contrast Microscopy

After 48 h of treatment with or without imine analogs of trans-resveratrol (aza-stilbenes) or with *trans*-resveratrol (6.25, 12.5 and 25 µM), the cell morphology and cell density of N2a cells were observed and photographed using a phase-contrast microscope (Axiovert 40 CFL, Zeiss, Jena, Germany) equipped with a digital camera (Axiocam lCm1, Zeiss).

### 4.5. Nuclei Staining with Hoechst 33342

The nuclear morphology of control (untreated cells) and treated cells cultured without or with AZA-ST or *trans*-resveratrol (6.25, 12.5 and 25 µM) for 48 h was characterized as previously described by fluorescence microscopy after staining with Hoechst 33342 (Sigma-Aldrich) (2 µg/mL) [23,48]. Cell deposits of about 40,000 cells were applied to glass slides by cytocentrifugation (5 min, 1500 rpm) with a cytospin 2 (Shandon, Cheshire, UK), mounted in Dako fluorescent mounting medium (Dako, Copenhagen, Denmark), and stored in the dark at 4 °C until observation. The morphological aspect of the cell nuclei was determined with an Axioskop fluorescent microscope (Zeiss). For each sample, 300 cells were examined.

### 4.6. Measurement of Plasma Membrane Permeability with Propidium Iodide

Propidium iodide (PI) was used to evaluate the plasma membrane permeability and cell death, as previously described [21]. This dye penetrates cells with damaged plasma membranes, which are considered dead cells [24]. After 48 h of treatment with or without AZA-ST 1a–g or *trans*-resveratrol, N2a cells (adherent and non-adherent cells) were stained with a PI solution (1 μg/mL of PBS) for 5 min at 37 °C, and then immediately analyzed on a BD Accuri™ C6 flow cytometer (BD Biosciences, San Jose, CA, USA). The red fluorescence was selected on a 630 nm band-pass filter, and 10,000 cells were acquired for each sample. Data analyses were performed using FlowJo v10.8.1 software (Tree Star Inc., Carrboro, NC, USA).

### 4.7. Flow Cytometric Evaluation of Transmembrane Mitochondrial Potential by Staining with Dihexyloxacarbocyanine Iodide (DiOC_6_(3))

The variation of the mitochondrial transmembrane potential (ΔΨm) was measured using 3,3′-dihexyloxacarbocyanine iodide (DiOC_6_(3)) (D273, Invitrogen/Thermo Fisher Scientific, Montigny le Bretonneux, France). This fluorochrome accumulates in the mitochondria proportionally to the ΔΨm value [49]. The higher the ΔΨm, the more the probe accumulates. After 48 h of treatment, adherent cells collected by trypsinization were pooled with non-adherent cells and stained with a solution of DiOC_6_(3) at 40 nM (15 min; 37 °C). The cells were immediately analyzed on a BD Accuri™ C6 flow cytometer (BD Biosciences). The loss of ΔΨm is indicated by a decrease in the intensity of the green fluorescence collected through a band pass filter of 520 ± 10 nm. For each sample, 10,000 cells were acquired, and the data were analyzed with the FlowJo v10.8.1 software (Tree Star Inc.). All assays were performed in triplicate.

### 4.8. Measurement of Reactive Oxygen Species Production with Dihydroethidium

Reactive oxygen species (ROS) production, including superoxide anion (O_2_^•−^) production, was measured by flow cytometry after staining with dihydroethidium (DHE) as previously described [21]. DHE is a dye that can freely diffuse across cell membranes; it is rapidly oxidized under the action of ROS to fluorescent ethidium. This latter exhibits an orange/red fluorescence (λEx max = 488 nm; λEm max = 575 nm). After 48 h of treatment, N2a cells (adherent and non-adherent cells) were stained with a 2 μM DHE solution for 15 min at 37 °C and immediately analyzed on a BD Accuri ™ C6 flow cytometer (BD Biosciences). The fluorescent signals of the DHE-stained cells were collected through a 580 nm band-pass filter, and 10,000 cells were acquired for each sample. Data analyses were performed using FlowJo v10.8.1 software (Tree Star Inc.).

### 4.9. Measurement of Mitochondrial Reactive Oxygen Species Production with MitoSox-Red

Mitochondrial production of ROS, including superoxide anion (O_2_^●−^), was quantified by flow cytometry after staining with MitoSox Red (Thermo Fisher Scientific). This positively charged probe accumulates in the mitochondria [50] and exhibits an orange/red fluorescence (λ Ex Max = 510 nm; λ Em Max = 580 nm). MitoSox Red stock solution was prepared at 5 mM in PBS and used at 5 μM in the cells. After cell treatments, adherent and non-adherent cells were pooled by trypsinization and incubated with MitoSox Red for 15 min at 37 °C. The fluorescent signals were collected through a 580 ± 20 nm band pass filter on a BD Accuri ™ C6 flow cytometer (BD Biosciences). For each sample, 10,000 cells were acquired. For each sample, 10,000 cells were acquired, and the data were analyzed with FlowJo v10.8.1 software (Tree Star Inc.). All assays were performed in triplicate.

### 4.10. Flow Cytometric Analysis of the Cell Cycle after DNA Staining with Propidium Iodide

Dead and live cells were collected in a tube by trypsination with a 1X trypsin solution and washed twice with 1X PBS. The cell pellet (around 4–5 × 10^6^ cells) was then suspended in 1 mL of ice-cold 80% ethanol to fix the cells, which were stored for 2 h at 4 °C [22]. Briefly, the fixed cells were then rehydrated by two washes in 1X PBS and resuspended in 300–400 µL of a solution containing 1X PBS, propidium iodide (IP, 80 µg/mL), and ribonuclease RNase A (200 µg/mL) for one hour at 37 °C. After incubation, stained cells were immediately analyzed by flow cytometry, and the red fluorescence was collected and quantified on a long-pass filter at 630 nm. The red fluorescence was measured on a linear scale considering 10,000 cells. The percentage of cells in the different phases of the cell cycle was determined using modeling software for cell cycle data (FlowJo_v10.8.1; Tree Star Inc., Carrboro, NC, USA).

### 4.11. Statistical Analysis

Statistical analyses of experimental results were performed using the GraphPad Prism 8.0 software (GraphPad Software, San Diego, CA, USA). The comparison of in vitro data was performed through an ANOVA test followed by a Bonferroni test, allowing multiple comparisons to evaluate any interaction. (*): *p* ≤ 0.05 was considered statistically significant.

## 5. Conclusions

In conclusion, our study brings new information on the cytotoxic properties of AZA-STs: (a) These molecules are cytotoxic and have a concentration range close to that of RSV; (b) like RSV, AZA-STs induce a strong oxidative stress both on whole cells and at the mitochondrial level; (c) unlike RSV, AZA-STs are either not or only weakly pro-apoptotic inducers; (d) unlike RSV, AZA-STs do not trigger an increase in the percentage of cells in the S phase of the cell cycle. In agreement with our previous study, which focused on the anti-oxidant properties of AZA-STs [15], our data bring new information supporting the idea that all AZA-STs synthesized have both different biochemical and biological properties. RSV is also known for its effects on immune cells [51] and on different signaling pathways involved in cell metabolism [52]. Additional studies of AZA-STs in these areas will deserve to be determined since cancer immunotherapy and metabolic therapy of cancer are future strategies of anti-tumor therapy.

## Data Availability

All data are available at the Institute of Molecular Chemistry of the University of Bourgogne (ICMUB UMR CNRS 6302; Dijon, France) and at Laboratory Bio-peroxIL (University of Bourgne/Inserm, Dijon, France).

## Sample Availability

Samples of the compounds aza-stilbenes (AZA-STs) and *trans*-resveratrol (RSV) are available upon request from the authors.
